# Green synthesis of iron oxide nanoparticles from Mexican prickly poppy (*Argemone mexicana*): assessing antioxidant activity for potential therapeutic use

**DOI:** 10.1039/d4ra07232d

**Published:** 2025-04-09

**Authors:** Aarti Nughwal, Ruchi Bharti, Ajay Thakur, Monika Verma, Renu Sharma, Annu Pandey

**Affiliations:** a Department of Chemistry, University Institute of Sciences, Chandigarh University Mohali Punjab 140413 India ruchi.uis@cumail.in; b KTH – Royal Institute of Technology, Fibre and Polymer Technology – Polymeric Materials, School of Chemical Science and Engineering Teknikringen 56-58 100 44 Stockholm Sweden annua@kth.se

## Abstract

This study presents an eco-friendly approach for synthesizing iron oxide nanoparticles using an extract from *Argemone mexicana* leaves, which function as reducing and stabilizing agents. The nanoparticles were thoroughly characterized using a range of techniques, including ultraviolet-visible (UV-vis) spectrophotometry, Fourier transform infrared spectroscopy (FTIR), scanning electron microscopy (SEM), X-ray diffraction (XRD), and zeta potential analysis. The synthesized Fe-NPs demonstrated notable antioxidant activity, as confirmed by assays involving 2,2′-azino-bis-(3-ethylbenzothiazoline-6-sulfonic) acid (ABTS) and 2,2-diphenyl-1-picrylhydrazyl (DPPH). The results highlight the significant antioxidant potential of these Fe-NPs. This research introduces a sustainable and innovative synthesis method for Fe-NPs, emphasizing their promising applications, particularly in fields related to antioxidant properties, as evidenced by the conducted antioxidant assays.

## Abstract 2

Green synthesis of iron oxide nanoparticles using *Argemone mexicana* leaf extract serves as a sustainable route. The biosynthesized nanoparticles exhibit strong antioxidant activity, demonstrating potential for therapeutic applications.

## Graphical abstract

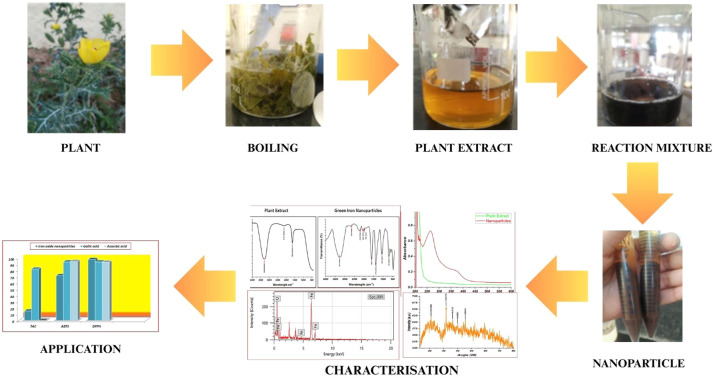

## Introduction

In nanotechnology, materials are deliberately modified through physical and chemical techniques to achieve specific properties tailored for particular applications.^1^ Nanoparticles, tiny materials less than 100 nanometers in size, differ significantly from bulk materials due to their unique thermal, optical, electronic, chemical, and physiological characteristics.^2,3^ These distinctive properties have driven the widespread use of nanoparticles in diverse fields, including medicine, chemistry, environmental science, energy, agriculture, electronics, information technology, and consumer products.^4^ This wide array of applications highlights the transformative impact of nanotechnology in advancing various scientific and technological fields.

Among the diverse range of nanomaterials, iron nanoparticles (INPs) have emerged as particularly intriguing due to their unique chemical and physical properties, including microwave absorption capacity, low toxicity, strong magnetism, and high catalytic activity.^5–7^ INPs are broadly categorized into three main groups: zero-valent iron nanoparticles, iron oxide hydroxide (FeOOH), and iron oxide nanoparticles such as maghemite (γ-Fe_2_O_3_), hematite (α-Fe_2_O_3_), and magnetite (Fe_3_O_4_).^8–11^ These nanoparticles have found applications in various fields, including drug delivery,^11^ magnetic targeting,^12^ hyperthermia,^13^ batteries,^14^ and pigments.^15^

Iron nanoparticles can be synthesized using either chemical or biological methods. The chemical approach, however, raises concerns regarding safety and cost due to the use of high-energy processes and hazardous chemicals, which are often flammable, toxic, and corrosive.^16^ As a result, green synthesis methods using biological samples have gained attention as a more environmentally friendly alternative, recognized for their simplicity and effectiveness. Among the various biological techniques for nanoparticle synthesis, plant extracts have been extensively explored.^17–20^ Fresh or dried plant materials, including leaves, fruits, bark, seeds, peels, and roots, have proven effective in the green synthesis of nanoparticles. Numerous studies in the literature describe methods where hazardous chemicals are replaced with plant-derived phytochemicals in the synthesis process.^21–27^ Given the advantages of plant extract-based synthesis, we have chosen to synthesize iron nanoparticles using *Argemone mexicana*.

**Fig. 1:**
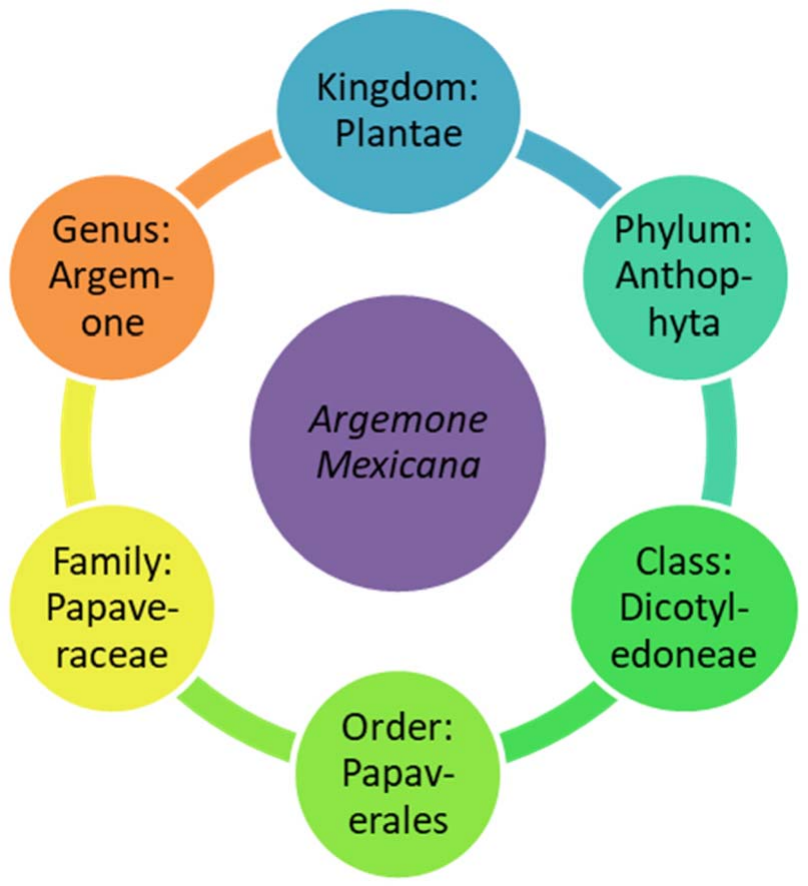
Classification of *Argemone mexicana*.^28^

*Argemone mexicana* falls under the taxonomic classification of kingdom: Plantae, phylum: Anthophyta, class: Dicotyledoneae, order: Papaverales, family: Papaveraceae, and the genus: *Argemone*^28^ (represented in Fig. 1).

*Argemone mexicana* is a promising candidate for green synthesis, particularly for iron nanoparticles, due to its unique botanical characteristics and biochemical richness. The plant is abundant in secondary metabolites such as alkaloids,^29,30^ flavonoids,^31,32^ and other phytochemicals,^33,34^ which are distributed throughout the plant. These compounds effectively reduce and stabilize agents during the green synthesis process, enhancing the efficiency and sustainability of nanoparticle production.

In this study, the leaf extract (LE) of *Argemone mexicana* was utilized to synthesize iron oxide nanoparticles (FeO-NPs). The LE's phytochemical richness enables it to function as both a stabilizer and a reducing bioagent during synthesis. This research focuses on the biosynthesis of INPs using *Argemone mexicana* LE and further investigates its antioxidant potential.

## Experimental

### Material and methods

All chemicals and solvents readily available commercially were used without any additional purification. Double-distilled water was utilized to prepare all aqueous solutions. Fresh leaves of *Argemone mexicana* were gathered from Maila Village in the Kangra district of Himachal Pradesh.

#### Preparation of plant extract

The leaves were gathered and cleansed using ordinary water and then significantly rinsed with deionized water (DW). Subsequently, 50 g of leaves were boiled in 1000 mL distilled water for 4 hours. The resulting solution was filtered using filter paper, yielding a 400 mL aqueous solution.

#### Preparation of aqueous solution of iron chloride

The aqueous solution of 0.1 M FeCl_3_ (anhydrous) was prepared by mixing 0.8 g in 50 mL of DW.

#### Synthesis of iron nanoparticles

**Fig. 2:**
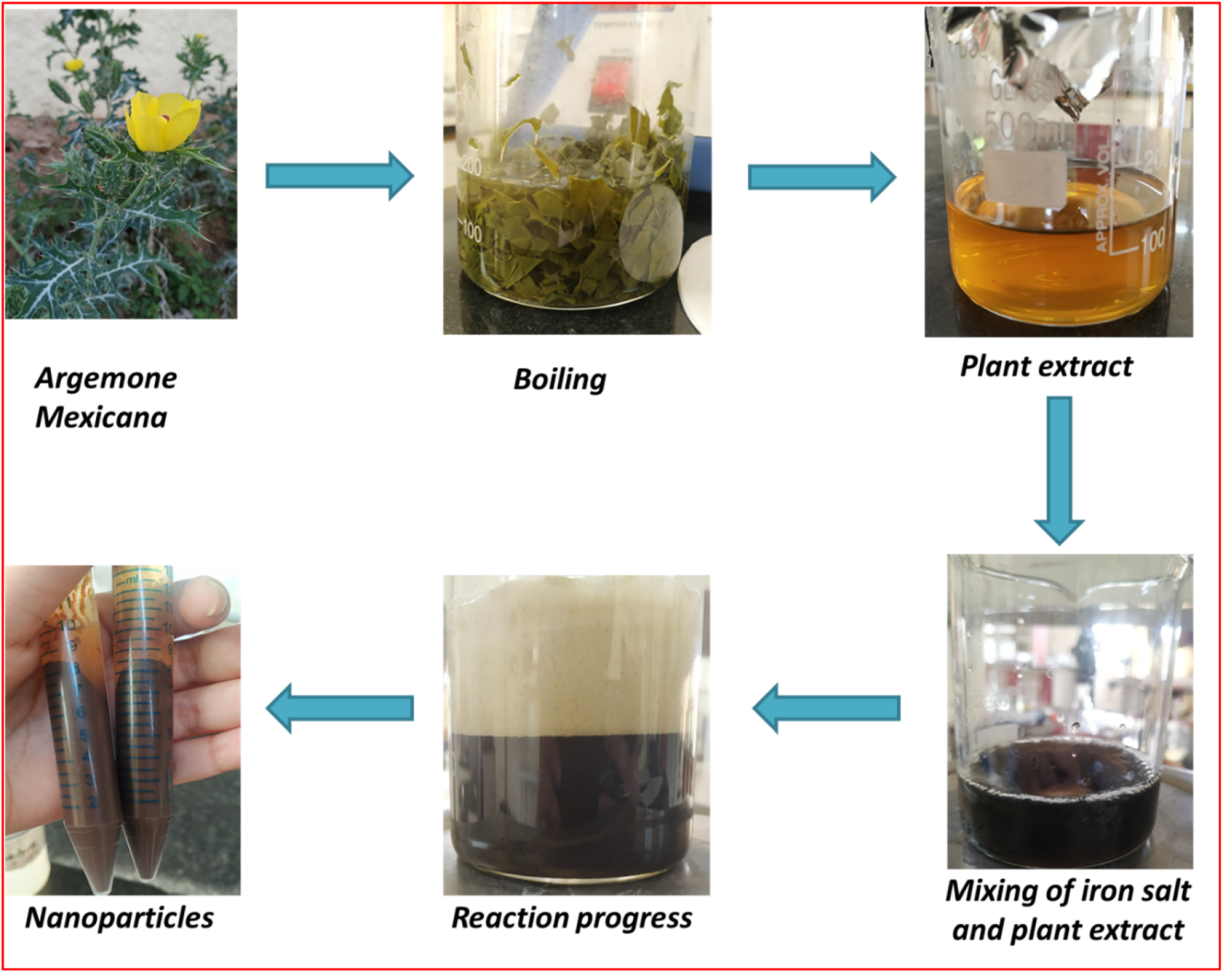
Green synthesis of iron nanoparticles.

In the experimental procedure, 50 mL of the plant extract was systematically combined with a gradual and controlled addition of a 0.1 M FeCl_3_ solution through a burette, ensuring a consistent drop rate. This process proceeded under continuous stirring for 2 hours at room temperature, as depicted in Fig. 2.

With the incremental addition of the plant extract to the metal salt solution, the solution's color transformed from orangish-yellow to a distinctive blackish-brown hue. This marked color change was visual evidence for FeO-NPs within the reaction mixture. Following the 2-hour reaction period, the resulting mix underwent centrifugation at 3000 rpm to facilitate the separation of nanoparticles from the solution. Subsequently, the obtained nanoparticles underwent a thorough washing process with distilled water and ethanol, repeated 2–3 times. This washing procedure was implemented to eliminate impurities and ensure the purity of the formed FeO-NPs. The systematic combination of these steps in the experimental protocol contributes to the controlled synthesis and purification of the nanoparticles, enhancing the reliability and reproducibility of the synthesis method.

#### Characterization of nanoparticles

The synthesized FeO-NPs were analysed using various instruments: UV-vis spectral analysis was carried out on a Shimadzu UV-1900 UV-vis spectrophotometer in the wavelength range of 200–800 nm at room temperature.

Fourier transform infrared (FT-IR) spectroscopy was performed using the PerkinElmer FT-IR Spectrometer with a scanning range of 4000–400 cm^−1^ with 50 scans to obtain the final spectrum.

The crystallographic properties were characterized using an analytical X'PERT PRO X-ray diffractometer (XRD) and data was collected over the range of 10° to 100° with a scanning speed of 0.5° min^−1^ and step size of 0.02°. Further, the determination of crystalline size of the synthesized FeO-NPs was conducted utilizing Scherrer's formula, expressed as follows:
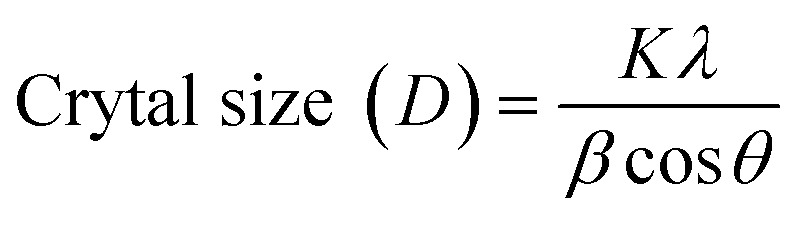
In this equation, *K* represents the shape factor (with a specific value of 0.94 in this context). *λ* signifies the incident X-ray wavelength of Cu-Kα radiation, precisely measured at 0.154252 nm. *β* denotes the full-width half maximum (FWHM), measured in radians. *θ* is Bragg's angle, expressed in radians.

Scherrer's formula is pivotal in determining the crystalline size of nanoparticles based on X-ray diffraction data. It establishes a relationship between the *D*, *λ*, the FWHM of diffraction peaks (*β*), Bragg's angle (*θ*), and the shape factor (*K*).

Scanning electron microscopy (SEM) was conducted using the SU8010 SERIES (HITACHI) and the analysis was done without any use of the additional conductive coating, relying on the intrinsic property of the sample. SEM was done at an accelerating voltage of 15 kV and working distance was maintained at 10.8 mm with a magnification set between 4500× to 12 000× in high vacuum mode to study the morphology, particle distribution and surface characteristics. Further for elemental analysis, Energy Dispersive X-ray Spectroscopy (EDX) was used in conjunction with the SEM.

For the DLS analysis (Litesizer 500 instrument), a quartz measurement cell was used and focus position was set automatically to −3.7 mm by the instrument. The measurement angle was set to backscatter, which was also controlled automatically. The target temperature remained at a constant 25.0 °C, and the equilibration time was kept to 30 seconds. DMSO was used as the solvent which showed a refractive index of 1.3751 and a viscosity of 1.9870 mPa s. The analysis followed a general model, and the cumulant model. 20 runs were processed manually, each taking 10 seconds, which were sufficient for obtaining reliable measurements.

Zeta potential (Litesizer 500 instrument) measurements were recorded using a Univette cell with DMSO as the solvent. The target temperature was set at 25.0 °C, while the solvent's refractive index measured 1.3751. With an equilibration time of just 20 seconds, the solvent viscosity was noted as 1.9870 mPa s, and the relative permittivity came to 42.42. Using the Smoluchowski model, the Henry factor was fixed at 1.5. The voltage, adjusted in automatic mode, was 27.2 V, and the analysis included 20 processed runs in manual mode. Before measurement the NPs was suspended in DMSO and ultrasonicated for 10 minutes to ensure uniform dispersion.

#### Antioxidant activity

The sample for the antioxidant study was prepared using dimethyl sulfoxide (DMSO) as the solvent. A concentration of 1 mg per mL was prepared and tests were performed in triplets to confirm the accuracy of the results. To assess its antioxidant potential, the DPPH (1,1-diphenyl-2-picryl-hydrazyl) radical scavenging assay, ABTS Radical Scavenging Assay and Total Antioxidant Capacity (TAC) was carried out following the method outlined by McDonald *et al.*,^35^ Khan *et al.*,^36^ and Biswas *et al.*^37^ respectively.

## Result and discussion

### UV-visible spectrophotometry

**Fig. 3:**
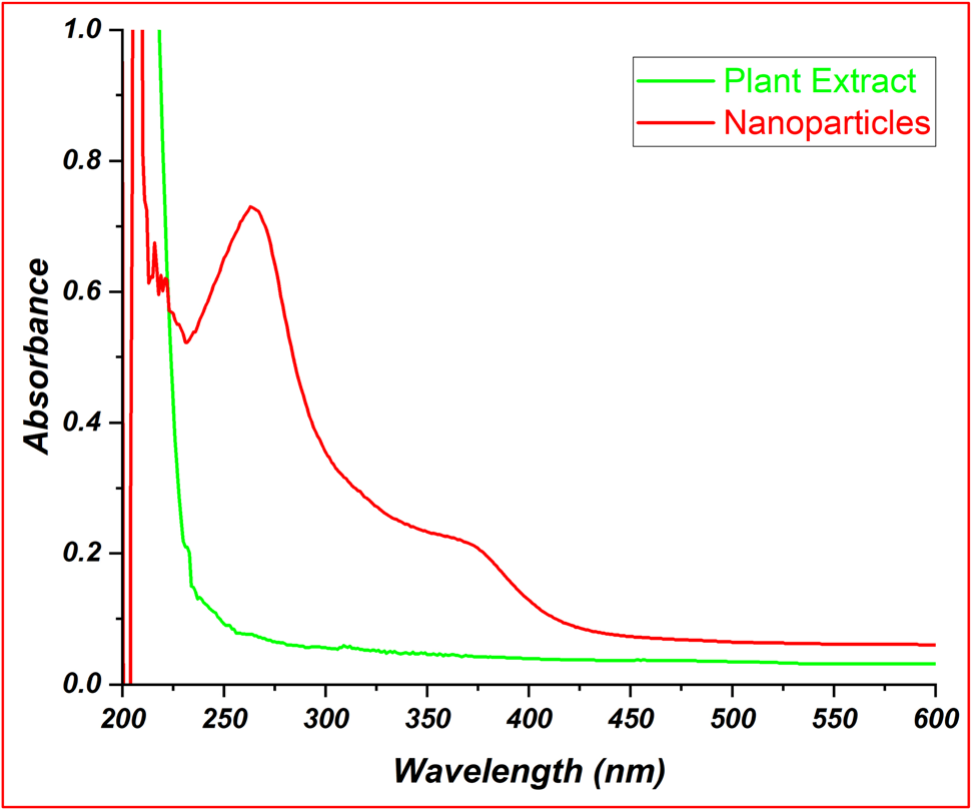
UV spectra of plant extract and synthesized nanoparticles.

UV-visible spectrophotometry is important in validating and characterizing the synthesized FeO-NPs. In Fig. 3, the plant extracts have no distinct absorption peak in the tested range, indicating the absence of strongly absorbing chromophores or conjugated systems in the extracts. The absence of the absorption peaks in *Argemone mexicana* plant extract is consistent to the findings reported by the Kailas *et al.*^38^ The absence of peak in plant extracts is in contrast to the UV absorption spectra of FeO-NPs which showed peaks at 250–300 nm and 350–400 nm (Fig. 3), serve as key indicators to validate the presence of iron oxide nanoparticles,^39^ showing defined absorption features indicative of synthesized nanoparticles.

The absorption data of the synthesized FeO-NPs obtained from the UV-visible spectrum was further compared with the literature to validate the finding. A study of Niraimathee *et al.* showed an absorption peak at 294 nm, which is associated with the presence of iron oxide nanoparticles (specifically Fe_3_O_4_). This peak corresponds to the formation of the nanoparticles, confirming their presence in the synthesized material. The FeO-NPs in this study show a similar peak in the 250–300 nm region, supporting the hypothesis that the synthesized nanoparticles are indeed iron oxide-based.^40^ In another study by Sharif *et al.*, the UV absorption band was observed in the 330–450 nm range, which is attributed to magnetic nanoparticles' absorption and scattering of light. Their study's absorption band at 410 nm indicates the formation of minimally agglomerated nanoparticles. This study further supports the synthesis of FeO-NPs using the *Argemone mexicana* plant extract.^41^

### IR spectroscopy

**Fig. 4:**
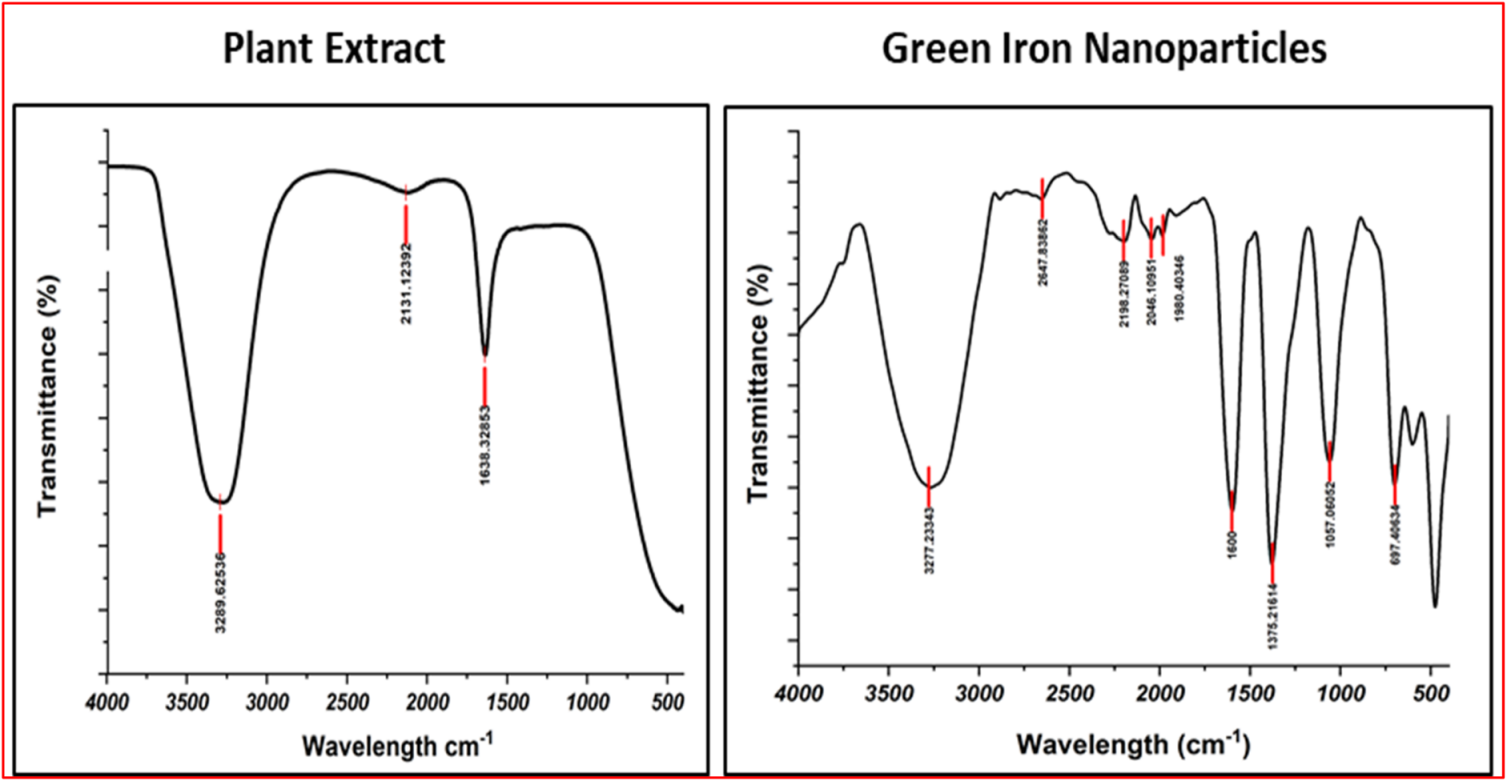
IR spectra of plant extract and green iron nanoparticles.

Infrared (IR) spectroscopy is an analytical technique which measures the absorption of IR radiation to analyze the vibrational modes of molecules and the resulting spectra serve as molecular fingerprints and provide the important insights about the structural composition of materials. Absorption patterns observed in spectrum correspond to specific molecular vibrations that are helpful in identification and characterization of the present functional groups within a sample. Obtained spectrum of the *Argemone mexicana* plant extract exhibits the prominent absorption peaks at 3289 cm^−1^, 2131 cm^−1^ and 1638 cm^−1^. The absorption peak at 3289 cm^−1^ suggested the presence of hydroxyl groups typically found in alcohols and phenols or –NH stretching vibrations (Fig. 4).^42^ Further the peak at 2131 cm^−1^ indicates stretching vibrations, suggesting the presence of N=C=S groups in the plant extract.^42^ The association with aromatic compounds was also observed due to the presence of the absorption band at 1638 cm^−1^ corresponds to C=C stretching vibrations.^43^ The collective presence of these functional groups containing nitrogen and oxygen functionalities is often involved in redox reactions. The presence of aromatic rings may further increase the reducing power of the extract.

While the IR spectra of the synthesized FeO-NPs confirmed the involvement of bioactive compounds in the nanoparticle synthesis process as significant changes was observed compared to the IR spectra of the extract alone. The absorption peak at 3289 cm^−1^ in the plant extract slightly shifted to 3277 cm^−1^ in the nanoparticle spectrum. This shift indicates the presence and involvement of –OH or NH_2_ group that gets absorbed onto the nanoparticle surface. The absorption band at 1638 cm^−1^ in the plant extract get shifted to the 1600 cm^−1^ (C=C stretching)^42^ in the nanoparticle spectrum, signifies a noteworthy alteration in the molecular environment of functional groups and attributed to the changes in the corresponding vibrational modes' chemical bonding or local electronic environments due to the coordination of aromatic compounds with the nanoparticle surface. Further, sharp peak observed at 1057 cm^−1^ corresponds to C–O stretching vibrations, indicating the involvement of organic acids, alcohols, or phenolic compounds from the plant extract.^43^ Another peak presence at 697 cm^−1^ is generally attributed to the Fe–O stretching vibrations and is characteristic of the iron oxide phase. This peak confirms the successful synthesis of iron oxide nanoparticles.^44,45^ The IR spectra of the synthesized iron oxide nanoparticles provide valuable insight into the role of the LE in the synthesis process. However, the exact mechanism remains inconclusive based solely on IR peaks as these observations provide indicative information. The IR spectra indicated a multifaceted role for the *Argemone mexicana* plant extract. Presence of specific functional groups potentially serves as a stabilizing agent and contributes to the capping of nanoparticles through its overall composition.

### Scanning electron microscopy (SEM)

**Fig. 5:**
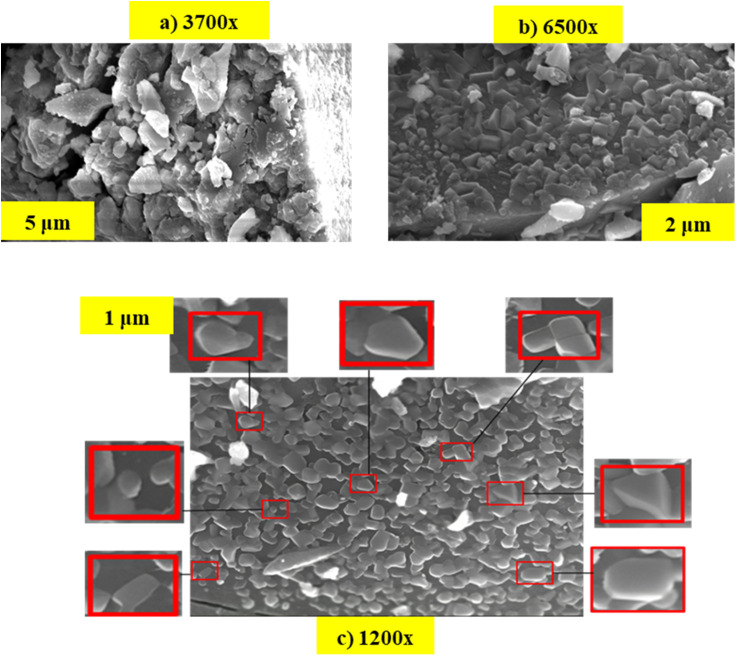
SEM images of synthesized iron oxide nanoparticles at various magnifications showing detailed morphological differences. (a) Image at 3700× magnification, scale bar represents 5 μm. (b) Image at 6500× magnification, scale bar represents 2 μm. (c) Image at 1200× magnification, scale bar represents 1 μm, highlighting the uniform particle distribution and size variation.

SEM is a powerful technique delivering highly detailed images that help in examining and understanding the surface morphology of materials at micro- to nanoscale resolution. Moreover, it allows for the visualization and analysis of three-dimensional surface topography such as particle size, shape, and surface roughness. Fig. 5 showcases the SEM image of the synthesized FeO-NpS at a magnifications of 3700–12 000× with a scale bar of 1–5 μm and used for the analysis of the nanoparticle's surface topography. In Fig. 5, as indicated in red boxes it is clear that the nanoparticles display a mix of shapes, predominantly hexagonal and irregular. A similar result was observed by Bouafia *et al.*^46^ and Arularasu *et al.*^47^ using the *Mentha pulegium* L and *K. alvarezii* plant extracts respectively. The nanoparticles are relatively uniform in size particles and appear to have sharp edges and distinct boundaries with some degree of agglomeration which is likely due to the magnetic interactions. The surfaces of the particles appeared smooth in the SEM image.

**Fig. 6:**
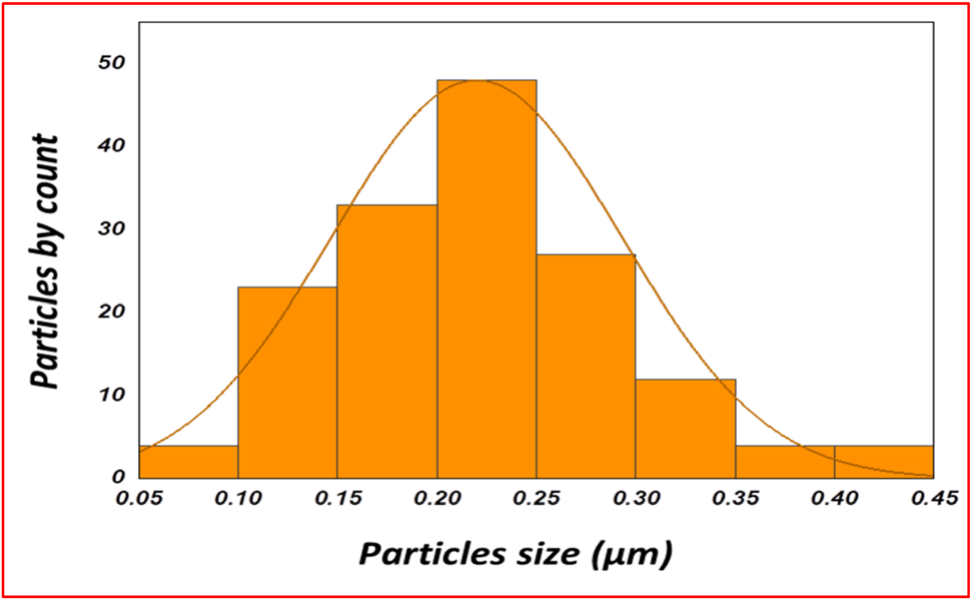
Particle size of synthesized nanoparticles.

Further, the average size was calculated using the Image J software and observed that the average length of the NPs was 0.220 ± 0.070 μm (Fig. 6).

### Energy dispersive spectroscopy (EDS)

SEM-EDS analysis of the synthesized NPs indicates the successful formation of iron oxide structures. Two distinctive peaks corresponding to oxygen (O) are prominent in the X-ray spectra, indicating the significant presence of oxygen in the synthesized iron nanoparticles. The first data set reports an elemental composition of 23.06% ± 0.59 for oxygen, constituting approximately 51.13% ± 1.32 of the total atoms. In contrast, the second data set shows a higher mass percentage of 76.94% ± 1.44 for oxygen, with an atom percentage of 48.87% ± 0.91 (shown in Table 1).

**Table 1:** SEM EDX analysis of the synthesized nanoparticles

Element	Line	Mass%	Atom%
O	K	23.06 ± 0.59	51.13 ± 1.32
Fe	K	76.94 ± 1.44	48.87 ± 0.91
Total		100.00	100.00

**Fig. 7:**
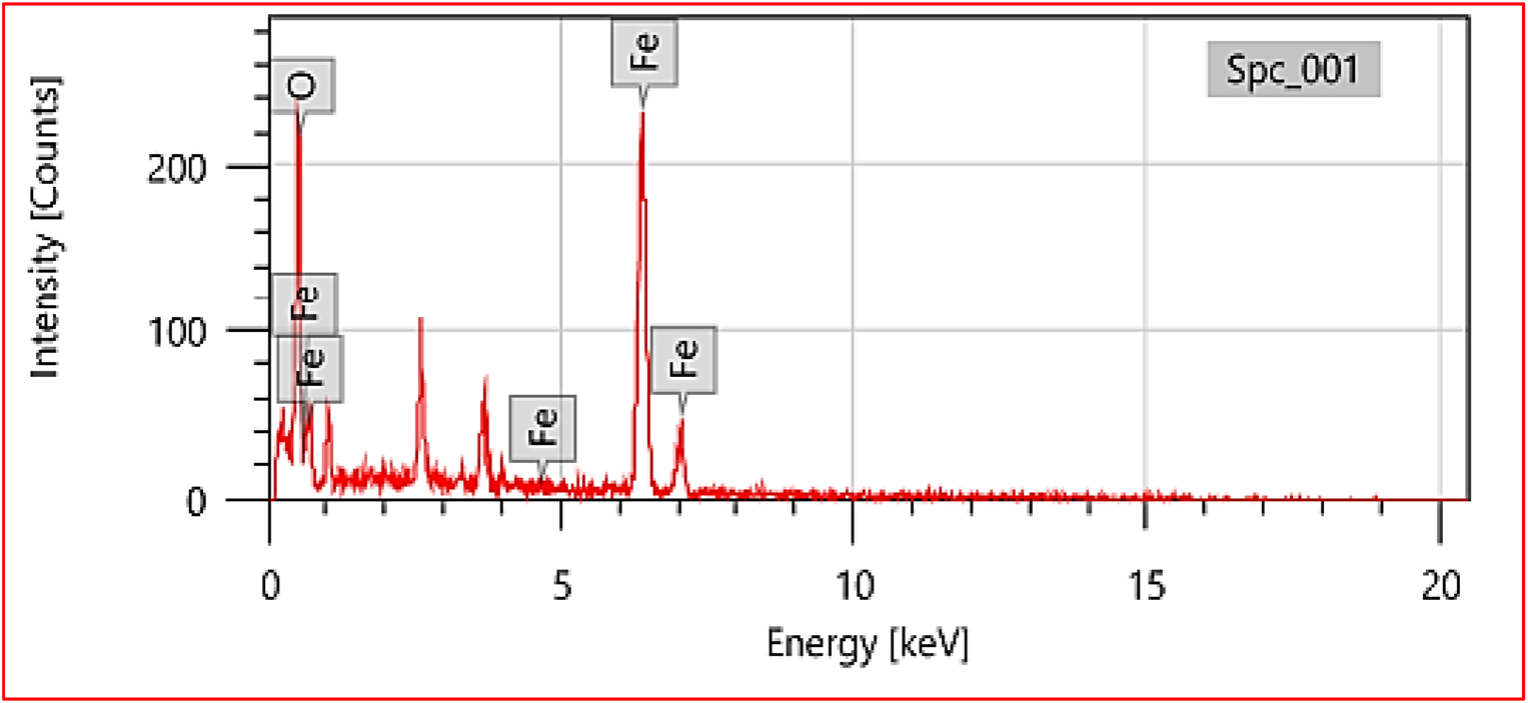
SEM EDX graph highlighting the presence of iron and oxygen.

These findings strongly suggest that the synthesized nanoparticles primarily comprise iron and oxygen (Fig. 7).

The consistency of the oxygen peak at the K-line in both spectra confirms the existence of oxygen in the synthesized FeO-NPs.

### X-ray diffraction spectroscopy

**Fig. 8:**
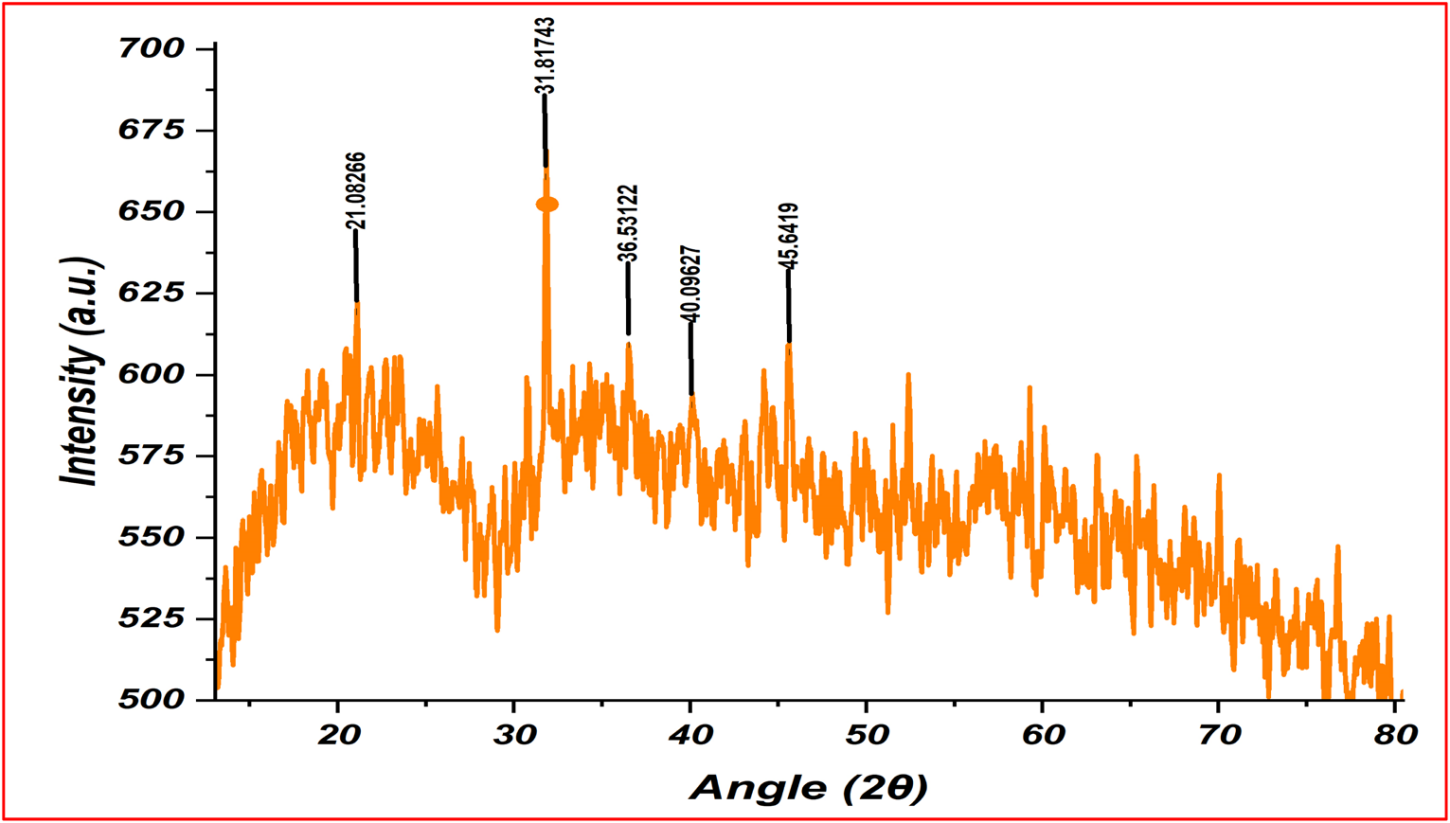
XRD pattern of synthesized iron oxide nanoparticles.

The XRD pattern analysis helps in determining the FeO-NPs nature as the absence of specific diffraction peaks in the XRD pattern indicates an amorphous structure which is characterized by a disordered atomic arrangement.^48^ Amorphous nature enhances the surface reactivity of FeO-NPs which makes amorphous FeO-NPs suitable for catalytic applications^49^ and drug delivery systems.^50^ In contrast well-defined lattice arrangement indicates the crystalline nature of the FeO-NPs and makes them suitable for applications in magnetic materials.^51,52^ XRD study provides valuable insights into the structural properties, which guide the selection of these FeO-NPs for specific applications based on their amorphous or crystalline nature. The XRD pattern of the synthesized FeO-NPs nanoparticles displayed in Fig. 8 reveals clearly defined peaks at 2*θ* values 21.08, 31.81, 36.53, 40.09 and 45.64. These finding aligns in close agreement with the reported 2*θ* values (18.97°, 29.81°, 35.24°, 39.53° and 48.30°) of FeO-NPs nanoparticles synthesized using the *Avicennia marina* extract by Karpagavinayagam *et al.*^44^ These observed peaks reflect the precise lattice arrangements within the crystalline structure and confirm the crystalline nature of the synthesized iron oxide nanoparticles.^44^ Further, the determination of the crystalline size of the synthesized FeO-NPs was done by utilizing Scherrer's formula as it establishes a relationship between the *D*, *λ*, *β*, *θ*, and *K*. The calculated average crystalline size derived from applying Scherrer's formula was found 15 nm of the synthesized FeO-NPs. This suggested that the plant extract obtained from the *Argemone mexicana* seems to be a powerful reductant and have a vital role in reducing the iron ions and contributing to the successful synthesis of crystalline FeO-NPs.

Further, the determination of the crystalline size of the synthesized FeO-NPs was done by utilizing Scherrer's formula as it establishes a relationship between the *D*, *λ*, *β*, *θ*, and *K*. The calculated average crystalline size derived from applying Scherrer's formula was found 15 nm of the FeO nanoparticles.

### Dynamic light scattering (DLS)

**Fig. 9:**
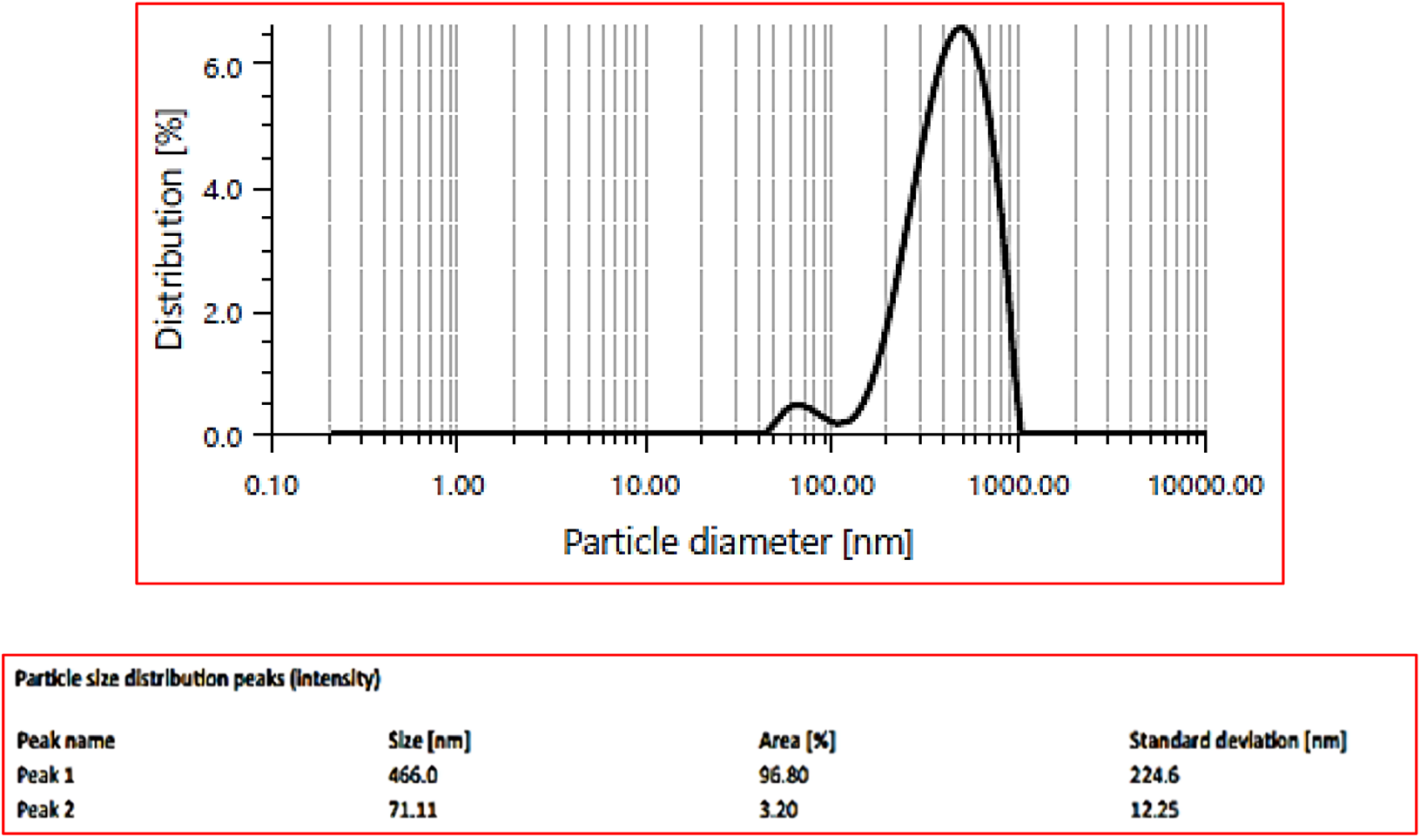
Hydrodynamic diameter of synthesized nanoparticles.

The average hydrodynamic diameter was determined to be 437.2 nm and the results presented in Fig. 9 indicated two distinct peaks in the particle size distribution, with corresponding size, area percentage, and standard deviation values. The DLS data also showed a polydispersity index (PDI) of 25.1% (0.251 in decimal form) polydispersity index which falls within the range of 0.1 to 0.4. This signifies that the particles are moderately polydisperse with moderate variation in particle size within the sample.^53^ In Fig. 9, peak 1 shows a size of 466.0 nm, which dominates the distribution with an area percentage of 96.80% with standard deviation of 224.6 nm. In contrast, peak 2 represents a much smaller fraction of the sample with a size of 71.11 nm and only 3.20% of the area with a smaller standard deviation of 12.25 nm. This data highlighted the particle uniformity in peak 2 compared to those in peak 1. Despite having a smaller size peak 2 provides valuable insight into the presence of finer particles in the sample. This data is consistent with the findings reported by the Demirezen *et al.*^54^ Further absolute intensity of 184 190.0 kcounts per s was observed which indicates the overall scattering behavior and reflects the degree to which they interact with light in a given environment. The diffusion coefficient which quantitatively measures the nanoparticle's mobility in fluid environments was found to be 0.5 μm^2^ s^−1^. In addition nanoparticles exhibits strong light absorption or scattering properties confirmed by the very low transmittance of 0.1%.

### Zeta potential

**Fig. 10:**
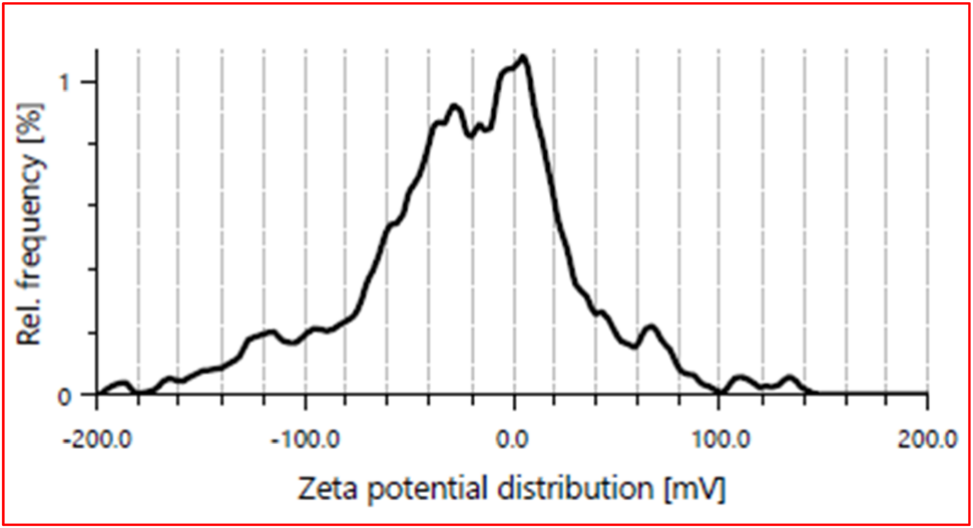
Zeta potential graph of synthesized nanoparticles.

This study provides a detailed analysis of the FeO-NPs zeta potential which on mean came out to be −12.0 mV (Fig. 10). The obtained zeta potential is consistent with the Lakshminarayanan *et al.* findings as they reported the synthesis of stable FeO-NPs from *Bauhinia tomentosa* extract with −16 mV zeta potential.^55^ Further standard deviation of 7.7 mV suggests a predominantly negative surface charge but is not completely uniform as having some degree of variability. Interestingly, the peak around 5.3 mV, highlights a somewhat consistent charge distribution that helps with colloidal stability. On top of that, the electrophoretic mobility was measured at −0.2270 μm cm V^−1^ s^−1^, which matches well with the negative zeta potential and shows how the material moves when exposed to an electric field. Optical properties show a mean intensity of 710.1 kcounts per s, and a filter optical density of 2.3094, giving insight into the material's light scattering and absorption characteristics. The conductivity value of 0.496 mS cm^−1^ and a low transmittance of just 0.2% point to potential applications where both electrical properties and light interaction are key.

### Antioxidant activity

In biological systems, antioxidants emerge as pivotal substances that effectively hinder the oxidation of molecules, thereby counteracting the adverse impacts of free radicals, ROS, and RNS. Their crucial involvement in maintaining cellular health lies in their capacity to neutralize oxidative stress, averting potential harm to DNA, proteins, and lipids. Beyond their fundamental role in scavenging free radicals, antioxidants wield a broader influence on overall well-being and are intricately linked to various health advantages. Among the advantages of antioxidants, one notable benefit is their potential protective effect against chronic diseases.^56,57^

In the present work, the iron oxide nanoparticles from *Argemone mexicana* demonstrated an impressive antioxidant activity compared with the iron-based nanoparticles reported in the literature as shown in Table 2. Nps derived using *Galium aparine* showed a DPPH scavenging activity of 85.43%, and Nps derived using *Phoenix dactylifera* exhibited a TAC value ranging from 77 to 180. Further *Asphodelus aestivus* based Nps displayed ABTS scavenging activity of 60.52 ± 0.01 and a modest DPPH scavenging activity of 3.48 ± 0.01. Other plants like *Curcumin*, *Aloe vera*, Green tea, and Ginger showed much lower activity than the aforementioned species.

**Table 2:** Comparison of antioxidant activity of the synthesized compounds with the literature

Sr. no.	Plant used	TAC	ABTS	DPPH	Ref.
1	*Argemonemexicana*	15.12 ± 001	71.70% ± 0.14	97.52%	Present work
2	*Galium aparine*	—	75.28%	85.43%	58
3	*Curcumin*	—	—	58.85%	59
4	*Aloe vera*	—	—	45.4%	59
5	Green tea	—	—	33%	59
6	Ginger	—	—	51.8%	60
7	*Phoenix dactylifera*	77–180	—	—	61
8	*Asphodelus aestivus*	—	60.52 ± 0.01	3.48 ± 0.01	62

**Fig. 11:**
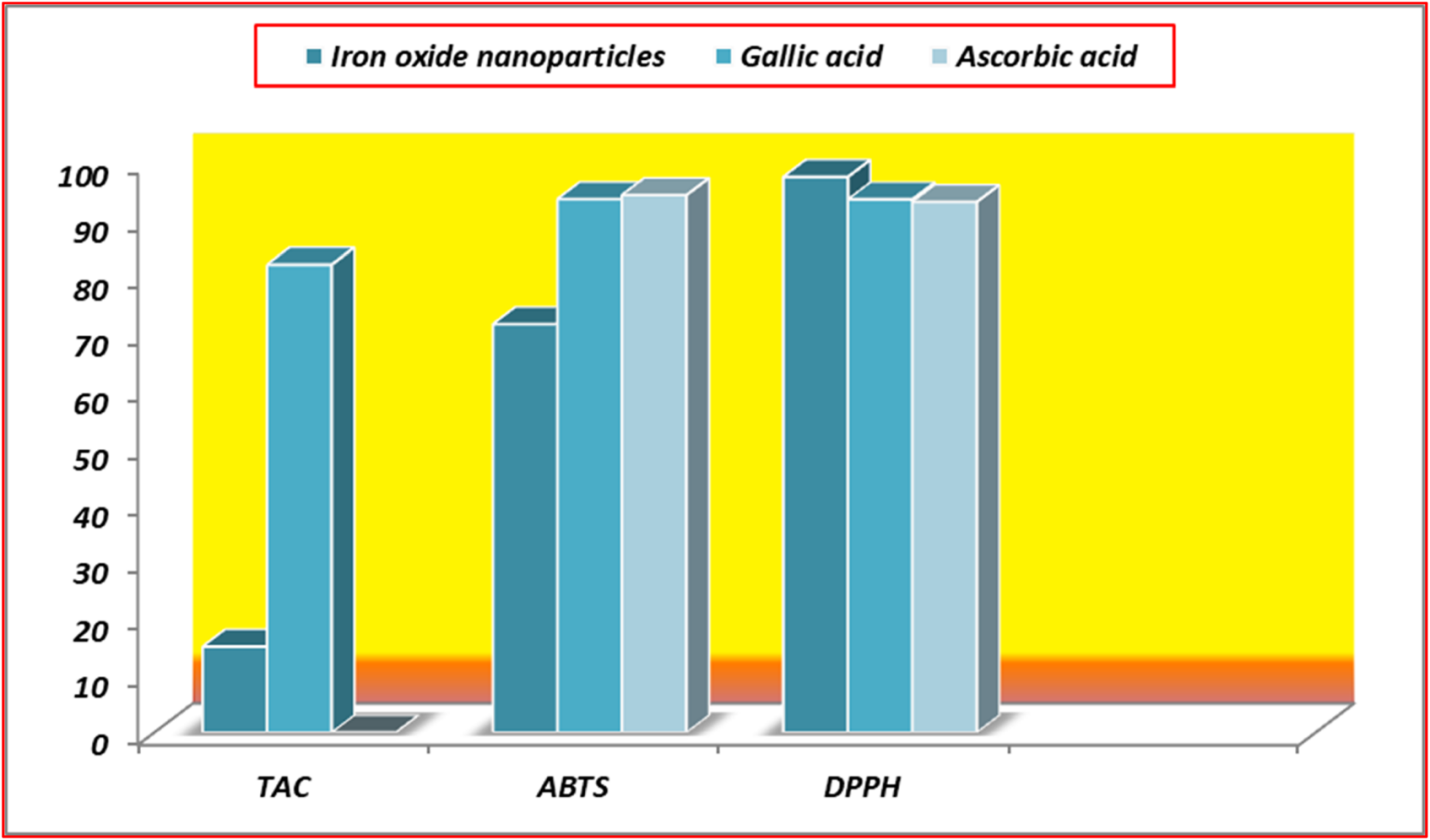
Antioxidant activities comparison.

Considering the role of antioxidants, the comprehensive evaluation of the antioxidant potential of iron nanoparticles (result presented in Table 2 and Fig. 11) was synthesized through an environmentally friendly method. It incorporated the concurrent application of three distinct assays. These assays, namely DPPH radical scavenging, TAC, and ABTS radical scavenging methods, were systematically performed to gauge the effectiveness of the synthesized nanoparticles in neutralizing free radicals.

In the TAC assay, the primary evaluation revolves around assessing the compounds' capability to reduce Mo^6+^ to Mo^5+^, especially under acidic conditions, measured spectrophotometrically at 695 nm.^63^ This reduction process acts as an indicator of the antioxidant potential within the tested compounds. The notable feature of this reaction is the observable color shift in the sample solution, transitioning from its initial state to a distinctly visible pale green hue. In our study, the synthesized nanoparticles exhibited activity with relatively low efficacy *i.e.* 15.12 ± 001, compared to gallic acid *i.e.* 82.05 (used as a standard).

Furthermore, the synthesized nanoparticles were assessed using the ABTS cation radical technique. This analytical approach is grounded in the principle that compounds endowed with antioxidant capabilities reduce the absorption at 734 nm by effectively scavenging the ABTS radicals.^64^ In the present study, noteworthy activity was observed (71.70 ± 0.14%) in comparison of gallic acid (94.93 ± 0.25%) and ascorbic acid (95.63 ± 0.15%) (both were used as a standard to compare the results), underscoring that all the synthesized nanoparticles exhibited significant antioxidant properties. This observation further emphasizes the potential utility of the synthesized nanoparticles as effective agents in combating oxidative stress and highlights their promising role in antioxidant applications.

The DPPH assay, analogous to the ABTS method, is a free radical technique employed to assess antioxidant potential. In this assay, DPPH radicals exhibit a vivid, deep blue color in alcoholic media. A noticeable transformation occurs upon introducing a substance with antioxidant properties, rendering the solution pale yellow or colorless. This change results from the swift reduction of DPPH radicals by active antioxidant substances, involving the acceptance of hydrogen or electrons. In this method, the absorbance of the sample was examined at a wavelength of 517 nm.^65^ Any decrease in the DPPH solution's absorbance highlights the compounds' scavenging potential. In our study, the synthesized iron nanoparticles showcased their antioxidant capabilities by exhibiting 97.52% inhibition, which is greater than the gallic acid (93.68 ± 0.41%) and ascorbic acid (92.92 ± 0.16%), offering valuable insights into their effectiveness in neutralizing free radicals through the DPPH reduction process.

The observed radical scavenging by the NPs can be ascribed to the presence of components with a bioactive nature within the extract. Consequently, the surface-functionalized FeO-NPs exhibit significant scavenging activity against free radicals. While these compounds show promise as potential antioxidant agents, further comprehensive evaluation is imperative to consider them for use as therapeutic agents. Continued research and exploration of their mechanisms of action and safety profiles will be crucial in harnessing the full therapeutic potential of these nanoparticles.

## Conclusions

In conclusion the environmentally friendly synthesis of FeO-NPs using *Argemone mexicana* leaf extract has shown to be quite effective. Various characterization techniques, such as UV-vis spectrophotometry, SEM, XRD, FTIR, DLS, and zeta potential analysis, were employed to get a clear picture of the physicochemical properties of nanoparticles. The zeta potential was found to be −12.0 mV suggesting good colloidal stability. Further, XRD studies showed the crystalline size of the nanoparticles averaged around 15 nm. SEM images revealed an average particle size of 0.220 ± 0.070 μm with uniformity in size and sharp edges with distinct boundaries and smooth surfaces. As far as their Antioxidant activity is concerned FeO-NPs showed good results with ABTS inhibition of 71.70% ± 0.14, and DPPH showing a 97.52% inhibition and have shown great promise as antioxidant agents. However more research is definitely needed to fully understand their therapeutic potential along with their mechanisms. This sustainable and efficient method provides a lot more promise in nanotechnology and biomedicine fields and can further open the doors for further exploration and development.

## Data availability

The data supporting this article have been included as part of the manuscript.

## Conflicts of interest

There are no conflicts to declare.
